# Unilateral Eosinophilic Fasciitis With Hand Involvement: A Case Report

**DOI:** 10.1002/ccr3.9613

**Published:** 2025-03-21

**Authors:** Christian Matthews, David Thomas, Luke Monteagudo, Jennifer Oberstar

**Affiliations:** ^1^ Department of Family Medicine and Community Health University of Minnesota Minneapolis Minnesota USA; ^2^ Division of Rheumatic and Autoimmune Diseases University of Minnesota Minneapolis Minnesota USA

**Keywords:** eosinophilic fasciitis, fibrosis, Shulman syndrome, systemic sclerosis

## Abstract

Eosinophilic fasciitis (EF) is a rare systemic connective tissue disease involving chronic inflammation of muscle fascia and subcutaneous tissue. While the underlying pathogenesis is poorly understood, prior publications have described classic findings to support this unusual diagnosis through clinical presentation, imaging, and histology. We report a unique case of eosinophilic fasciitis in a 24‐year‐old male with a predominantly asymmetric presentation and related hand involvement. Key features of the physical exam, labs and MRI imaging led to the diagnosis, and the patient improved with steroids and eventually transitioned to steroid sparing therapy. This case report highlights an unusual presentation of eosinophilic fasciitis, reviews classic diagnostic criteria and underscores a situation when it may be reasonable to avoid full thickness skin biopsy and opt for early treatment.

**Summary**

- Eosinophilic fasciitis is a rare disease of chronic inflammation of muscle fascia and subcutaneous tissue.
- We present a case of asymmetric presentation with hand involvement, which is unique to our knowledge.
- Clinical presentation, work‐up with labs and MRI, and treatment are discussed for the education of clinicians who may encounter this disease, for which early intervention is key.

## 1 Introduction

Eosinophilic fasciitis (EF) is a rare scleroderma‐like disorder involving chronic inflammation of muscle fascia and subcutaneous tissue. The disease typically has symmetric involvement of the extremities and is characterized by skin induration, elevated inflammatory markers, and peripheral eosinophilia [1, 2, 3]. Most cases are diagnosed in the fourth to fifth decade of life. There is no difference in prevalence among genders. Although the underlying etiology of eosinophilic fasciitis is uncertain, it has been associated with several possible triggers including trauma, intense exercise, hematologic disorders, radiation therapy, and medication exposures. Potential factors associated with the diagnosis of EF include trauma due to intense exercise prior to symptom onset which occurs in 30%–46% of patients [4, 5, 6]. Hematologic disorders such as aplastic anemia, myeloproliferative disorders, and multiple myeloma are present in 10% of EF patients and thus may be associated [7]. EF cases have been documented after radiation therapy, burns, hemodialysis, and medication exposures (statins, phenytoin, ramipril, subcutaneous heparin, and immune checkpoint inhibitor therapy) [7, 8, 9, 10, 11] To a lesser extent, autoimmune disorders such as thyroid disease, systemic lupus erythematosus, and Sjogren's and infections, most notably borrelia burgdorferi, have been associated as well.

We report a unique case of eosinophilic fasciitis in a 24‐year‐old male with asymmetric presentation with related hand involvement which to our knowledge has not previously been described.

## 2 Case History and Exam

**FIGURE 1:**
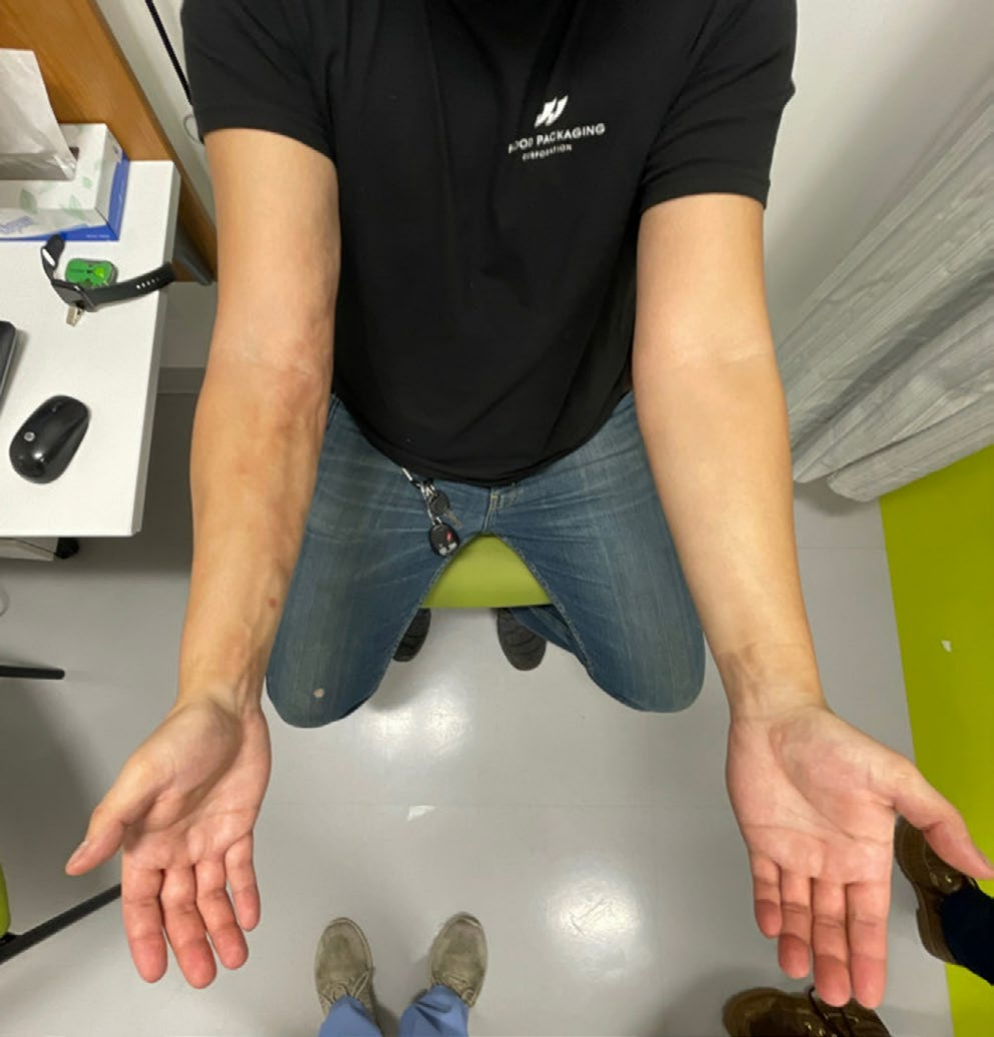
Demonstrating unilateral presentation photograph taken at initial rheumatology visit (11 months after symptom onset), depicting progressive presentation of symptoms.

**FIGURE 2:**
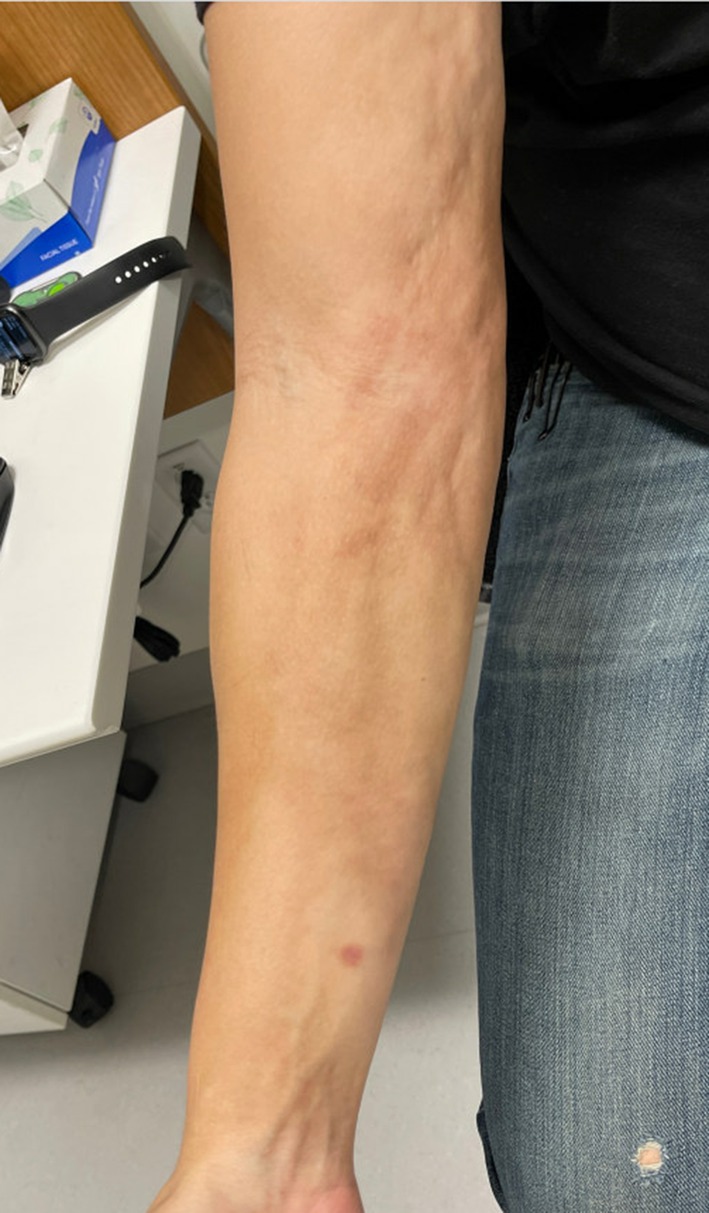
Demonstrating peau d'orange skin texture. Photograph taken at initial rheumatology visit (11 months after symptom onset), depicting progressive presentation of symptoms.

**FIGURE 3:**
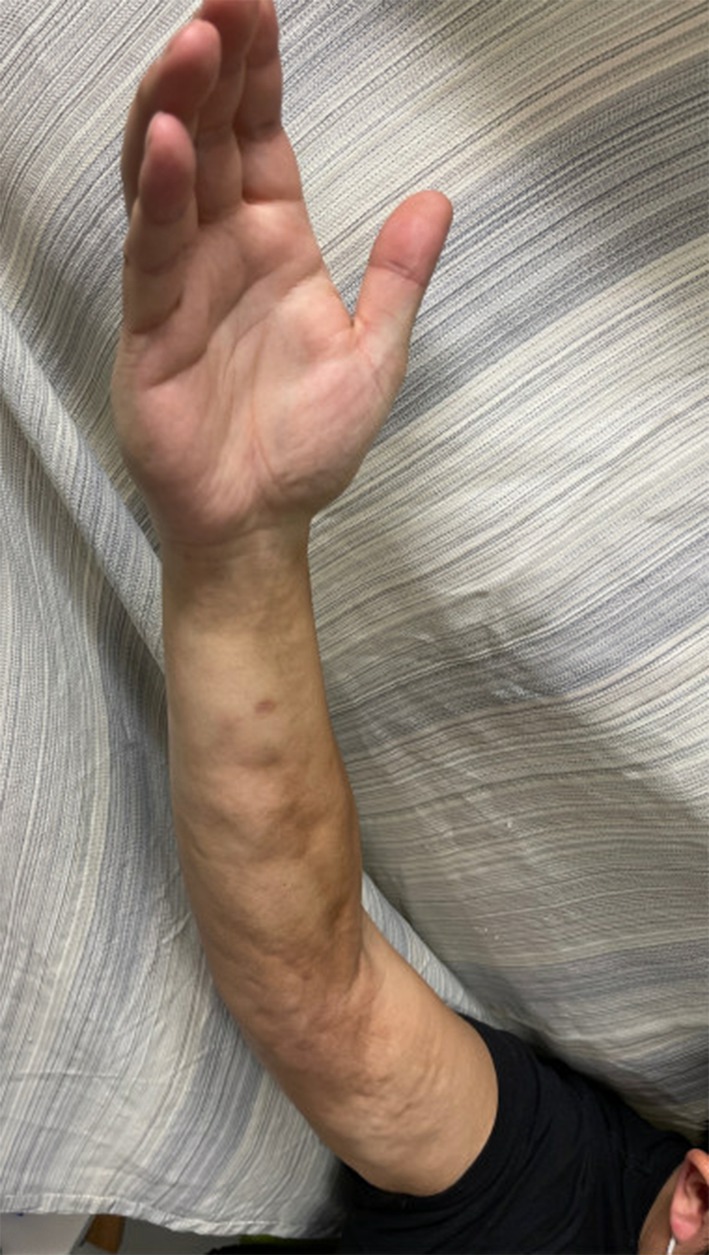
Demonstrating unusual hand involvement as well as classic “groove sign”. Photograph taken at initial rheumatology visit (11 months after symptom onset), depicting progressive presentation of symptoms.

A 24‐year‐old otherwise healthy right‐hand dominant male presented to his primary care provider with right forearm pain and swelling with new onset arthralgias for 6 months. The patient worked as an operator at a packaging facility for 7 years where he would frequently carry large rolls of wrap over his right shoulder. Six months prior to symptom onset, he had transitioned to a less manually intensive, managerial role. He first noticed discoloration of his right upper extremity with an increasing feeling of painful tension within the right forearm (Figures 1, 2, 3). He concurrently noticed similar tension and swelling in both lower extremities as well as decreased right wrist mobility. His primary care provider who noted 2+ non‐pitting edema in the lower extremities and an area of tightness at the right forearm that was not tender to palpation.

The patient had not been prescribed medications or been exposed to supplements or known toxins prior to the onset of symptoms. He did not smoke, drink, or use illicit drugs.

## 3 Differential Diagnosis

The differential diagnosis at this point is quite broad, but must include a breadth of dermatologic and rheumatologic localized and systemic disorders that affect the skin as well as underlying muscle and connective tissues. The main considerations with this presentation other than eosinophilic fasciitis include systemic sclerosis (SSc), localized scleroderma, and other scleroderma‐like disorders. In more unique cases, one must consider reactions to chemical exposure, toxic oil syndrome, and graft‐versus‐host‐disease.

## 4 Results

Lab work‐up at that time revealed an elevated C‐reactive protein (CRP, 20.3 mg/L), elevated absolute eosinophil count (1500 eosinophils/μL) and a positive anti nucleic acid antibody (ANA) with a 1:80 titer and speckled pattern. Cyclic citrullinated peptide antibody, rheumatoid factor antibody, lyme antibody, thyroid stimulating hormone and erythrocyte sedimentation rate (ESR) were within normal limits, and lower extremity ultrasounds were negative for deep vein thrombosis (DVT), thrombophlebitis or cyst. He was subsequently referred to sports medicine for further evaluation.

**FIGURE 4:**
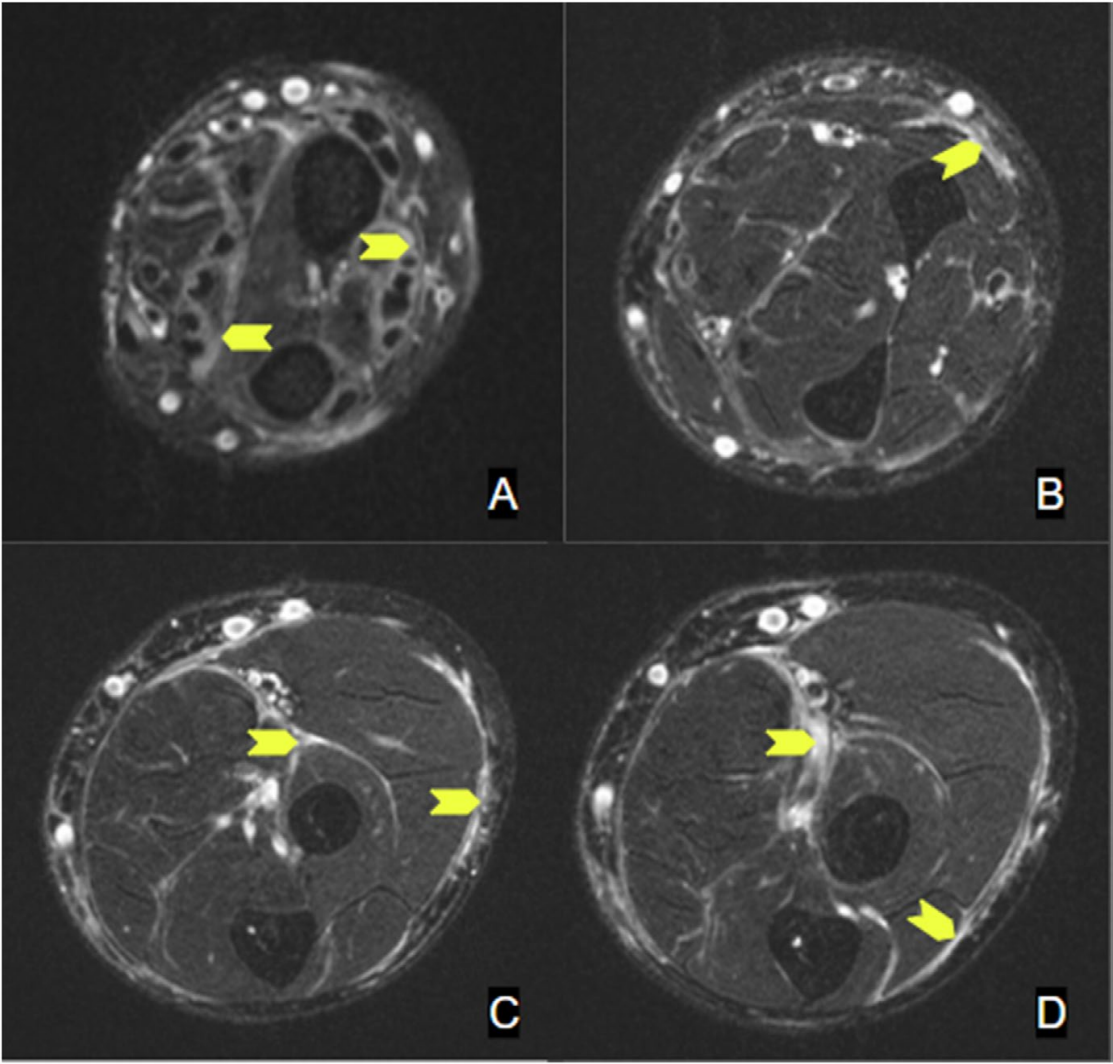
Left forearm axial T2‐weighted MRI. (A–D) distal to proximal demonstrating increased signal in the subcutaneous fascia.

**FIGURE 5:**
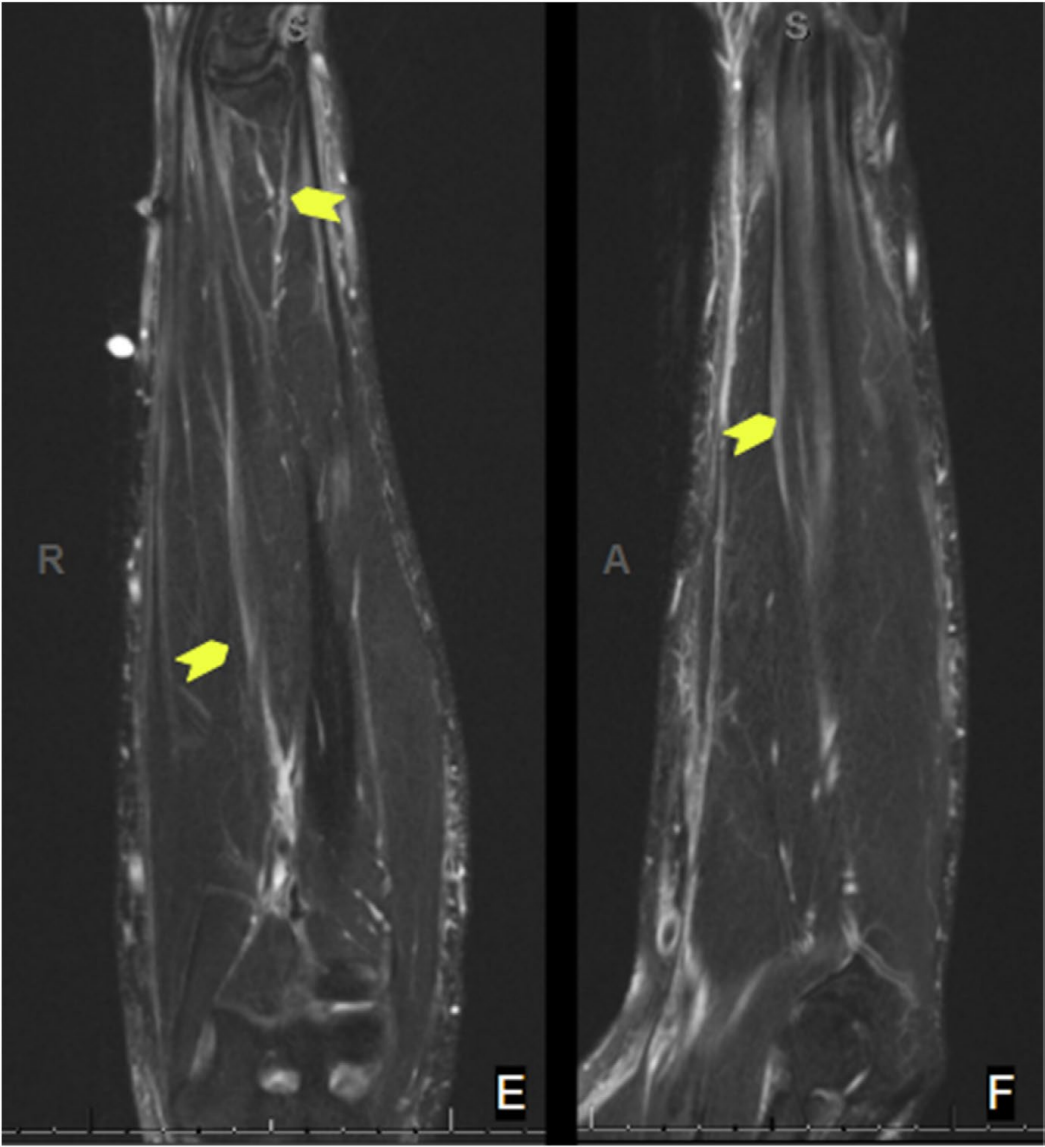
Coronal (E) and sagittal (F) left forearm axial T2‐weighted MRI images demonstrating increased signal in the subcutaneous fascia.

When seen in the sports medicine clinic 4 months later (10 months after symptom onset), the patient reported that he had attempted stretching, chiropractic massage, cupping, and electrotherapy to the extremities without significant improvement. He was experiencing a new inability to flex the fingers of the right hand alongside new morning stiffness and arthralgia in bilateral knees and wrists. Lab testing revealed worsening absolute eosinophil count (2200 eosinophils/μL) and increasing CRP (60.2 mg/L). Upper extremity dopplers were negative for DVT. A right forearm magnetic resonance imaging (MRI) revealed diffuse multi compartment deep layer fasciitis without muscle involvement (Figures 4 and 5). Given the progression of his disease and knowledge that early intervention improves clinical outcomes, he was immediately started on prednisone 40 mg daily.

At his initial rheumatology appointment (11 months after symptom onset), the patient's physical exam was notable for sclerodactyly of the fingers on the right hand, right forearm venous furrowing consistent with peau d'orange texture (Figure 2) and the “groove sign” (Figure 3), extending from the right bicep to right wrist. He had limited flexion and extension of the right elbow, right wrist, and fingers of the right hand due to the tightness and thickening of the skin. He had normal nailfold capillaries and no evidence of telangiectasias, calcinosis, or mucosal disease. Review of systems was negative for dry eyes, dry mouth, dysphagia, shortness of breath, lower extremity edema, or triphasic color change of the extremities consistent with Raynaud's phenomenon.

## 5 Outcome and Follow‐Up

His prednisone was adjusted to a high dose (60 mg daily) with a prolonged taper. He also started weekly oral methotrexate and folic acid. He was prescribed daily pantoprazole for ulcer prophylaxis and sulfamethoxazole‐trimethoprim for Pneumocystis pneumonia prophylaxis while on high‐dose steroids. Complement levels, gamma globulins and antibodies to RNA polymerase III, PM/Scl‐100, Scl‐70, antinuclear ribonucleoprotein, Smith, centromere, double stranded DNA as well as Anti Sjögren's Syndrome A and B antibodies all returned within reference range.

By 6 weeks of treatment, his symptoms had significantly improved and so, biopsy was not pursued per shared decision making. Unfortunately, due to the GI side effects of the methotrexate therapy over the course of 3 months, the patient was switched to hydroxychloroquine. He also required a slightly increased dose of prednisone during his taper due to ongoing symptoms during this transition.

## 6 Discussion

Our case describes a patient with a unique presentation of presumptive eosinophilic fasciitis. The combination of his relatively young age, predominantly unilateral skin findings and hand involvement has not been adequately reported in the literature. Otherwise, his presentation is classic for eosinophilic fasciitis based on his laboratory and radiologic findings [4, 8, 11, 12].

EF is most commonly characterized by its exam findings. Edema which progresses to induration of the skin and deeper perimuscular fascial planes are characteristic early in disease. The edema is typically symmetric, though unilateral disease can occur. Erythema and peau d'orange texture are common secondary skin findings. Though the rash and initial presentation may appear similar to systemic sclerosis (SSc), the skin changes in EF typically occur on the arms and legs, neck, and trunk versus the fingers, hands, and face involvement in SSc. In addition, SSc commonly affects the fingers and is associated with Raynaud's phenomenon, whereas this would be uncommon in EF [12, 13].

The most specific skin finding of EF is the “groove sign” (Image 3). To elicit the groove sign, the patient should elevate the affected edematous limb, which will result in visible indentation along the course of the superficial veins. This occurs as a result of fibrosis of the connective tissue surrounding blood vessels with sparing of the epidermis and superficial dermis. When the affected arm is elevated, decreasing the peripheral venous pressure, the vein becomes visible as an accentuated depression [8, 14].

Myalgias, arthralgias, and muscle weakness are common. Arthritis and neuropathies have been noted in a minority of patients. Compared to other conditions such as SSc, restrictive lung disease, pleural effusions, pericarditis, proteinuria (glomerular and tubular) have been described inconsistently with EF [8, 15].

The majority of patients will have peripheral blood eosinophilia early in the disease, though it may be transient. The degree of eosinophilia does not correlate with severity of disease. Over 50% of patients will have elevated ESR, CRP, and polyclonal hypergammaglobulinemia. Serum ANA and CK are typically normal, even for those with myalgias [4, 16].

The diagnosis of EF has historically been confirmed with a full thickness skin‐to‐muscle biopsy. Findings of lymphoplasmacytic infiltrate with histiocytes and eosinophils are typical and differentiate this from systemic sclerosis and other scleroderma‐like disorders. Although fascial biopsy has been considered the gold standard for diagnosing EF, MRI has been increasingly used for diagnosis and monitoring of treatment. The prototypical MRI findings include fascia inflammation or thickening, often seen as increased T2 signal in the subcutaneous and deep fascia and enhancement on fat‐suppressed T1 images after contrast [17, 18, 19, 20]. For our case, after shared decision making with the patient, biopsy was not pursued due to the classic findings on exam, labs, and MRI, knowing that earlier treatment would be of the most importance.

EF is treated with systemic corticosteroids, although there are no randomized trials of EF therapies. Currently, the best proposed approach is treatment with medium to high‐dose corticosteroids, followed by a slow taper depending on clinical response, which may take weeks to months. For those with severe, incomplete response, or recurrent disease, higher doses of corticosteroids along with an immunosuppressive drug may be used. Methotrexate is most often used, however azathioprine, cyclophosphamide, cyclosporine, and immuno‐biologic agents such as IVIG, TNF‐alpha, and rituximab have been used [6, 7, 8, 11, 17]. Up to 75% of patients respond to corticosteroid therapy and about half achieve remission. Early intervention and response is not always predictive of future skin or articular outcomes; however, recurrent or treatment resistant disease can be associated with chronic skin thickening and localized soft tissue contractures [20, 21].

## 7 Summary

EF is a rare inflammatory disorder that is often underdiagnosed but can be recognized by classic findings in the patient history, labs, imaging as well as full thickness skin biopsy. Because of the disfiguring and potentially debilitating symptoms of the disease, it is a valuable diagnosis for providers to consider within a differential, especially since early treatment can minimize severe clinical sequelae.

## Author Contributions

**Christian Matthews:** conceptualization, data curation, investigation, writing – original draft, writing – review and editing. **David Thomas:** conceptualization, data curation, investigation, writing – original draft, writing – review and editing. **Luke Monteagudo:** conceptualization, data curation, funding acquisition, project administration, supervision, writing – review and editing. **Jennifer Oberstar:** conceptualization, data curation, funding acquisition, project administration, supervision, writing – review and editing.

## Consent

The patient was provided the case report in advance of journal submission with ample time to provide feedback. The patient denied any additional commentary regarding patient perspective, and all patient feedback was incorporated into the case report prior to journal submission. After review of the case report, a consent form was reviewed with the patient, signed and is available on request.

## Conflicts of Interest

The authors declare no conflicts of interest.

## Data Availability

Data sharing is not applicable to this article as no new data were created or analyzed in this study.

## References

[1] L. E. Shulman , “Diffuse Fasciitis With Eosinophilia: A New Syndrome?,” Transactions of the Association of American Physicians 88 (1975): 70–86. 1224441

[2] H. Long , G. Zhang , L. Wang , and Q. Lu , “Eosinophilic Skin Diseases: A Comprehensive Review,” Clinical Reviews in Allergy and Immunology 50 (2016): 189–213, 10.1007/s12016-015-8485-8. 25876839

[3] A. V. Marzano and G. Genovese , “Eosinophilic Dermatoses: Recognition and Management,” American Journal of Clinical Dermatology 21 (2020): 525–539, 10.1007/s40257-020-00520-4. 32394361

[4] D. Lebeaux and S. Damien , “Eosinophilic Fasciitis (Shulman Disease),” Best Practice & Research. Clinical Rheumatology 26 (2012): 449–458, 10.1016/j.berh.2012.08.001. 23040360

[5] L. Bischoff and C. T. Derk , “Eosinophilic Fasciitis: Demographics, Disease Pattern and Response to Treatment: Report of 12 Cases and Review of the Literature,” International Journal of Dermatology 47, no. 1 (2008): 29–35, 10.1111/j.1365-4632.2007.03544.x. 18173597

[6] Y. Adachi , Y. Mizutani , E. Shu , H. Kanoh , T. Miyazaki , and M. Seishima , “Eosinophilic Fasciitis Associated With Myositis,” Case Reports in Dermatology 7 (2015): 79–83, 10.1159/000381845. 26034478 PMC4448070

[7] S. Lakhanpal , W. W. Ginsburg , C. J. Michet , J. A. Doyle , and S. B. Moore , “Eosinophilic Fasciitis: Clinical Spectrum and Therapeutic Response in 52 Cases,” Seminars in Arthritis and Rheumatism 17, no. 4 (1988): 221–231, 10.1016/0049-0172(88)90008-x. 3232080

[8] D. Lebeaux , C. Francès , S. Barete , et al., “Eosinophilic Fasciitis (Shulman Disease): New Insights into the Therapeutic Management From a Series of 34 Patients,” Rheumatology (Oxford) 51 (2012): 557–561, 10.1093/rheumatology/ker366. 22120602

[9] I. Pinal‐Fernandez , A. Selva‐O' Callaghan , and J. M. Grau , “Diagnosis and Classification of Eosinophilic Fasciitis,” Autoimmunity Reviews 13, no. 4–5 (2014): 379–382, 10.1016/j.autrev.2014.01.019. 24424187

[10] R. Hariman , P. Patel , J. Strouse , M. P. Collins , and A. Rosenthal , “Development of Eosinophilic Fasciitis during Infliximab Therapy for Psoriatic Arthritis,” Case Reports in Rheumatology 2016 (2016): 4, 10.1155/2016/7906013. PMC4879227 27293946

[11] H. S. Al‐Ghamdi , “Vigorous Exercise‐Induced Unilateral Eosinophilic Fasciitis: Rare and Easily Misdiagnosed Subtype,” International Journal of Clinical and Experimental Pathology 13, no. 7 (2020): 1739–1744. 32782698 PMC7414499

[12] A. Primitivo , N. Madeira , D. Lopez , and D. Afonso , “Eosinophilic Fasciitis (Shulman Disease) With Clinical, Imaging and Pathological Correlation,” BML Case Reports 14 (2021): e246151. 10.1136/bcr-2021-246151 PMC8719132 34969795

[13] R. Fruchter , D. R. Mazori , and A. N. Femia , “Groove Sign of Eosinophilic Fasciitis,” Journal of Clinical Rheumatology 23 (2017): 169, 10.1097/RHU.0000000000000524. 28277348

[14] M. B. Jacobs , “Eosinophilic Fasciitis, Reactive Hepatitis, and Splenomegaly,” Archives of Internal Medicine 145 (1985): 162–163. 3970632

[15] J. H. Chun , K. H. Lee , M. S. Sung , and C. J. Park , “Two Cases of Eosinophilic Fasciitis,” Annals of Dermatology 23, no. 1 (2011): 81–84, 10.5021/ad.2011.23.1.81. 21738370 PMC3120006

[16] D. R. Mazori , A. N. Femia , and R. A. Vleugels , “Eosinophilic Fasciitis: An Updated Review on Diagnosis and Treatment,” Current Rheumatology Reports 19 (2017): 74, 10.1007/s11926-017-0700-6. 29101481

[17] M. Ronneberger , R. Janka , G. Schett , et al., “Can MRI Substitute for Biopsy in Eosinophilic Fasciitis?,” Annals of the Rheumatic Diseases 68 (2009): 1651–1652, 10.1136/ard.2008.103903. 19748919

[18] S. J. Moulton , M. J. Kransdorf , W. W. Ginsburg , A. Abril , and S. Persellin , “Eosinophilic Fasciitis: Spectrum of MRI Findings,” AJR. American Journal of Roentgenology 184, no. 3 (2005): 975, 10.2214/ajr.184.3.01840975. 15728627

[19] F. Baumann , P. Brühlmann , G. Andreisek , B. A. Michel , B. Marincek , and D. Weishaupt , “MRI for Diagnosis and Monitoring of Patients With Eosinophilic Fasciitis,” AJR. American Journal of Roentgenology 184 (2005): 169–174. 15615969 10.2214/ajr.184.1.01840169

[20] R. Tull , W. D. Hoover, 3rd , J. F. de Luca , W. W. Huang , and J. L. Jorizzo , “Eosinophilic Fasciitis: A Case Series With an Emphasis on Therapy and Induction of Remission,” Drugs in Context 7 (2018): 212529, 10.7573/dic.212529. 30302114 PMC6172017

[21] E. Zuelgaray , S. Chevret , M. Jachiet , et al., “Trunk Involvement and Peau d'orange Aspect Are Poor Prognostic Factors in Eosinophilic Fasciitis (Shulman Disease): A Multicenter Retrospective Study of 119 Patients,” Journal of the American Academy of Dermatology 88, no. 1 (2023): 160–163, 10.1016/j.jaad.2020.11.009. 33188872

